# Health impact assessment of waste management facilities in three European countries

**DOI:** 10.1186/1476-069X-10-53

**Published:** 2011-06-02

**Authors:** Francesco Forastiere, Chiara Badaloni, Kees de Hoogh, Martin K von Kraus, Marco Martuzzi, Francesco Mitis, Lubica Palkovicova, Daniela Porta, Philipp Preiss, Andrea Ranzi, Carlo A Perucci, David Briggs

**Affiliations:** 1 Department of Epidemiology, Regional Health Service, Lazio, Via Santa Costanza 53, 00198, Rome, Italy; 2 Department of Epidemiology and Biostatistics, Imperial College, London, UK; 3 World Health Organization, Regional Office for Europe, Copenhagen, Denmark; 4 World Health Organization, Regional Office for Europe, Rome, Italy; 5 Department of Environmental Medicine, Slovak Medical University, Bratislava, Slovakia; 6 Institute of Energy Economics and the Rational Use of Energy, University of Stuttgart, Germany; 7 Environmental Health Reference Centre, Regional Agency for Environmental Prevention of Emilia Romagna, Modena, Italy

## Abstract

**Background:**

Policies on waste disposal in Europe are heterogeneous and rapidly changing, with potential health implications that are largely unknown. We conducted a health impact assessment of landfilling and incineration in three European countries: Italy, Slovakia and England.

**Methods:**

A total of 49 (Italy), 2 (Slovakia), and 11 (England) incinerators were operating in 2001 while for landfills the figures were 619, 121 and 232, respectively. The study population consisted of residents living within 3 km of an incinerator and 2 km of a landfill. Excess risk estimates from epidemiological studies were used, combined with air pollution dispersion modelling for particulate matter (PM_10_) and nitrogen dioxide (NO_2_). For incinerators, we estimated attributable cancer incidence and years of life lost (YoLL), while for landfills we estimated attributable cases of congenital anomalies and low birth weight infants.

**Results:**

About 1,000,000, 16,000, and 1,200,000 subjects lived close to incinerators in Italy, Slovakia and England, respectively. The additional contribution to NO_2 _levels within a 3 km radius was 0.23, 0.15, and 0.14 μg/m^3^, respectively. Lower values were found for PM_10_. Assuming that the incinerators continue to operate until 2020, we are moderately confident that the annual number of cancer cases due to exposure in 2001-2020 will reach 11, 0, and 7 in 2020 and then decline to 0 in the three countries in 2050. We are moderately confident that by 2050, the attributable impact on the 2001 cohort of residents will be 3,621 (Italy), 37 (Slovakia) and 3,966 (England) YoLL. The total exposed population to landfills was 1,350,000, 329,000, and 1,425,000 subjects, respectively. We are moderately confident that the annual additional cases of congenital anomalies up to 2030 will be approximately 2, 2, and 3 whereas there will be 42, 13, and 59 additional low-birth weight newborns, respectively.

**Conclusions:**

The current health impacts of landfilling and incineration can be characterized as moderate when compared to other sources of environmental pollution, e.g. traffic or industrial emissions, that have an impact on public health. There are several uncertainties and critical assumptions in the assessment model, but it provides insight into the relative health impact attributable to waste management.

## Background

Waste collection, transport, processing, and disposal are important for both environmental and public health reasons. Different strategies have been proposed to reduce, reuse, recycle, recover energy and finally dispose of municipal solid waste (MSW) [1,2] but the environmental impacts of these strategies are controversial [3]. Several studies of the possible health effects on populations living in proximity of landfills and incinerators have been published [4] and both landfills and incinerators have been associated with some reproductive and cancer outcomes. However, the results of these studies are either inconclusive or contradictory. Consequently, there is controversy over the possible health implications of waste management policies [5] and both policy makers and the public require more information on the likely health impacts (and importantly, the associated nature and extent of the uncertainties).

**Figure 1:**
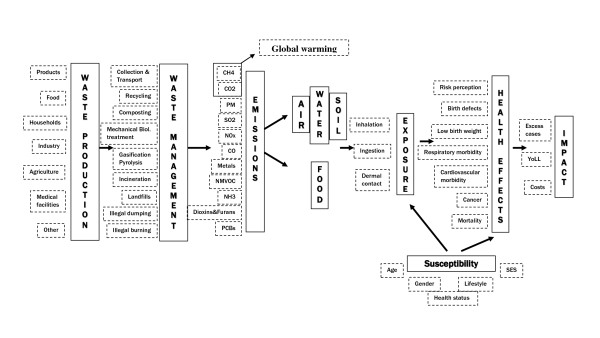
**The full chain approach - from waste production to health effects**.

Within the EU-funded INTARESE project [6], we aimed at assessing potential exposures and health effects arising from municipal solid wastes, from generation to disposal, or treatment (see Figure 1 to appreciate the full chain of the waste problem). The background of the project is that policy cannot be served solely by using traditional risk assessment methods. Instead, the assessment needs an approach that recognises the complexity of the systems involved, and integrates the different information, knowledge, analytical methods and tools needed to approach the way these complex systems behave. It was this need that motivated the term "integrated environmental health impact assessment" (IEHIA), whose rationale, guidance and instruments are illustrated in the toolbox available on the web (http://www.integrated-assessment.eu).

We performed a diagnostic assessment for three EU countries (Italy, Slovakia and England) considering the baseline situation in 2001 in order to assess the health burden or risk attributable to specified factors. This initial approach might be of interest for ranking different risks to health in terms of their overall burden of disease, and thus for prioritising policy action. For reasons of feasibility in our study, not all the aspects illustrated have been considered here (e.g. waste transport, occupational factors, greenhouse gases, risk perception etc) and they could be part of a more complete and exhaustive exercise. However, we aimed to establish a baseline scenario that can be useful in the future for prognostic assessment of different policy options.

Each step in the exercise has several uncertainties that should be considered in the overall evaluation. For this reason, we have tried to systematically state the level of confidence we had using the scale of calibrated terminology used by the IPCC [7]: very high (at least 9 out of 10), high (about 8 out of 10), moderate (about 5 out of 10), low (about 2 out of 10), and very low (less than 1 out of 10). Such calibrated levels of confidence can be used to characterize uncertainty based on expert judgment of the correctness of a model, an analysis or a statement. The approach was also applied in the systematic review we conducted on the topic [4].

## Methods

The assessment followed the steps involved in the "full chain" approach, illustrated in Figure 1.

### Waste Generation and Management

Current waste management data were collected from country-specific environmental agencies. The Italian Institute for Environmental Protection and Research (ISPRA) [8] provided a database of the incinerators operating during the period 2001-2007. In addition, a detailed census of the 52 incinerators operating in 2005 was made available by a national research institute [9]. Detailed data were also provided by the regional environmental authority of Emilia Romagna for all eight incinerators located in that region. From all these sources, we were able to identify the 40 incineration plants operating in 2001, obtain their geographical coordinates and get specific information on years of operation, number of lines, fumes capacity (Nmc/h), stack height (m), stack diameter (m), exit velocity (m/s), emission rate (m^3^/s), and exit temperature (°C). In a few cases, when the information on technical characteristics was missing, it was approximated using information from other plants with similar characteristics.

The ISPRA provided a database of landfills in Italy (a total of 619 in 2001) with information of the total capacity and wasteland filled per year. Geographical coordinates of the landfills were available for only five (of twenty) regions (Piedmont and Emilia Romagna (North), Tuscany and Abruzzi (Centre), and Campania (South)) for a total of 118 landfills. For the rest of the country, we assumed that the characteristics (sex, age and socioeconomic status) of people around the 501 missing landfills were similar to those of the entire sample of the 118 sites studied.

For Slovakia, information on the number of incinerators handling municipal waste in 2001 was obtained from the Slovak Environmental Agency (SEA). There were two incinerators for MSW in 2001, geo-coded and with detailed information on technical characteristics, obtained from managing companies. At the end of 2001, there were 165 active landfills for municipal wastes. The list of landfills, by region, was available from the website of the Slovak Ministry of Environment [10], with geographical coordinates, capacity and starting year obtained from the SEA. Out of 165 active landfills in 2001, 121 were geocoded. We assumed that the characteristics (sex, age and socioeconomic status) of people around the 45 missing landfills were similar to those of the 121 studied sites.

For England, we evaluated all 11 municipal waste incinerators operating during 2001. Data on emissions of toxic substances (Tonnes/annum) and location (x- and y-coordinates) were obtained from the Environment Agency (EA). Wherever possible, more specific data (e.g. stack height, stack diameter, emission rate, etc) were obtained from the waste companies' websites. Where no data could be found, we applied an average from the known incinerator data.

Data for all regulated landfill sites in England and Wales was obtained from the EA. No data for 2001 was available because of changes in the regulations. In 2001 landfill sites came under a different directive and were not required to report to the EA under the Pollution Prevention and Control (PPC) Regulations. The EA advised using the 2006 landfill data instead as a good indicator for the 2001 situation [11]. The 2006 dataset contains information about 242 regulated landfill sites (e.g. geographic Cartesian coordinates, atmospheric releases).

### Population data by gender, age and socioeconomic status

Population data at the smallest unit of aggregation for the 2001 census were available for the census blocks in Italy (about 100-200 (mean 162, sd 223) inhabitants per unit) and Slovakia (about 700-800 (mean 785, sd 1318) inhabitants). For England, census population data were disaggregated to postcode areas (mean 41, sd 37). For each census block in Italy, a deprivation index was available [12]. It used census information that represents various aspects of deprivation: education, occupation, home ownership, family composition and nationality. An algebraic combination of these factors was used to create an index of socioeconomic position by census block, with the corresponding population distributed in quintiles, ranging from very well off (level 1) to very underprivileged (level 5). For Slovakia, an index of socioeconomic position was derived from the following census variables: education (proportion of population with university, secondary, basic or no education), proportion of families with children, proportion of employed among 16-64 year olds, house type (house or flat), and house ownership. Again an index per census block was distributed in quintiles, ranging from very well off (level 1) to very underprivileged (level 5). For England, the Carstairs score [13], which is based on four census variables (lack of car ownership, unemployed head of the household, low social class and overcrowding) was applied as the deprivation index. The Carstairs score was available at the smallest census area, the output areas (OAs). By means of a point-in-polygon analysis the Carstairs score was transferred from the OA to each postcode. As in Italy and Slovakia, the Carstairs score in England was divided into 5 quintiles, 1 being the most affluent to 5 being the most deprived.

We used the distance from the point source (landfill site and/or incinerator) to estimate the exposed population. We decided to use the 3 km surrounding incinerators [14] and 2 km surrounding landfill sites [15] as the likely limit of the dispersion of emissions. For both incinerators and landfills, we defined increasing radial distances (1, 2, and 3 km) from the centre (the formal address of the plant) and evaluated the census blocks (or the postcode districts) that matched these locations. In several cases, the distribution of census blocks did not precisely fit the circle and the borders were tailored in order to more precisely count the population. The validity of the method has been evaluated using individually geocoded data of the resident population in four areas of Emilia Romagna (Italy) and considering the border of the plant area rather than the formal address: the error range was between 1 and 10%.

### Local air dispersion modelling

Local air dispersion modelling was used to calculate increased pollutant concentrations (particulate matter, PM_10_, and Nitrogen dioxide, NO_2_) within 3 km of the waste incinerators. Dispersion modelling for incinerators was based on the national information on incineration census, actual waste throughput data and meteorological data. We have used the Atmospheric Dispersion Modelling System (ADMS-Urban) [16] for modelling dispersion at the local scale for 40 incinerators in Italy, 2 in Slovakia and 11 in England. Meteorological data requirements include temperature (°C), wind speed (m/s), wind direction (°), precipitation (mm), cloud cover (oktas), relative humidity (%), boundary layer height (m), and surface sensible heat flux (W/m^2^). We have used official meteorological data available from the nearest meteorological station. Usually 2001 meteorological data were used.

For PM_10 _and NO_2 _we have used emission rates based on national limits derived from EU legislation in 2001, namely daily emission rates of 10 and 200 mg/Nm^3^, respectively. However, since actual emissions could be estimated from Italy and England, we conducted an additional analysis using this measurement data [17,18].

A number of incinerators in both Italy and England were located on hilly terrain. ADMS-Urban contains a hill module that takes into account the surrounding terrain when modelling the dispersion. Terrain data was therefore obtained for both these countries. For England the Ordnance Survey PANORAMA ™ Digital Terrain Model was used to obtain surface heights for 50 × 50 m cells up to 10 km away from 8 of the 17 incinerators. For Italy the terrain data was collected from the Italian Environmental Protection Agency for 35 of the 40 incinerators.

ADMS air pollution dispersion model provided "contours" of additional concentrations of PM_10 _and NO_2 _for the incinerators. These output files (one per country) have been transferred into the GIS system. The population database at the smallest available unit (i.e. census block or postcode district) for the given radius of 3 km has been added to the GIS as another data layer. Using an overlay function in GIS, the population data was combined with the air pollution concentration data with a grid of 200 meters. In this way, different statistics regarding population-weighted exposure levels have been estimated according to gender, age and socioeconomic status.

### Exposure-response relationships

Following a systematic review of the literature [4], we chose to use the excess risk values reported by Elliott et al. [14] of cancer for incinerators, and of congenital malformations and low birth weights [15] for landfills. Cancer incidence between 1974 and 1987 among over 14 million people living near 72 solid waste incinerator plants in Great Britain were studied [14]. The excess risk estimate for living within 3 km of an incinerator for all cancers combined was 3.5% (95%CI = 3-4%). However, Elliott et al. point out that there was an indication of residual confounding from socioeconomic status and a concern of misdiagnosis among registrations and death certificates for liver cancer. These aspects lowered our overall confidence in the results and we rated the level of confidence of the risk estimates for cancer as "moderate".

In the national study conducted by Elliott et al. [15] on 9,565 landfill sites in Great Britain, operational at some time between 1982 and 1997, statistically significant increased risks were found for all congenital malformations, neural tube defects, abdominal wall defects, surgical correction of gastroschisis and exomphalos, and low and very low birth weight in residents within 2 km of the sites. Although several alternative explanations, including ascertainment bias, and residual confounding could not be excluded in the study, it provides quantitative effect estimates: the relative risk for congenital anomalies was 1.02 (99% CI = 1.01-1.03) and 1.06 (99% CI = 1.052-1.062) for low birth weight. Again, on the basis of the systematic review [4] our level of confidence in these relative risks was "moderate".

Linear and no-threshold exposure-response functions related to the long-term effects on mortality from PM_10 _and NO_2 _have been derived from the extensive existing reviews of epidemiological and toxicological data [19]. We assumed a linear relationship between the air pollutants and associated health effects as most epidemiological studies on large populations have been unable to identify a threshold concentration below which ambient air pollutants has no effect on morbidity and mortality. The following values were used:

### Background health statistics

Background gender-age specific cancer incidence data for the three countries were retrieved [20-22] together with national mortality statistics [22-24]. Prevalence of congenital malformations at birth was derived from the Annual Report (data for 2000) of the International Clearinghouse for birth defects monitoring system [25] for Italy and England, and from The Statistical Office of the Slovak Republic [24] for Slovakia.

### Time frame of the assessment

Health impacts were estimated for the period 2001-2050, assuming that the incinerators operating in 2001 will be operating until 2020 - a realistic assumption given that these plants are usually in operation for a long time. The choice of 2050 ensures that the time period under consideration is long enough to account for chronic effects. For incinerators, cancer incidence "attributable" to exposure before 2001 ("past exposure") was estimated (burden of disease non-modifiable by policy) as it is likely that it will continue to appear until 2050. In addition, cancer incidence "attributable" to exposure during 2001-2020 was estimated ("current exposure") as these effects could be, at least in part, prevented. In addition, Years of Life Lost (YoLL) were estimated as attributable to current exposure (2001-2020) to PM_10 _and NO_2 _in the cohort of 2001 residents followed up to 2050.

For landfills operating in 2001, we assumed that the emissions will last up to 2030 (an assumption supported by the available knowledge that landfilled biodegradable waste starts to emit biogas a few years after deposit and continues to do so for several decades) and the health effects, in terms of congenital anomalies and low birth weight, are constant throughout this period. It was assumed that there would be no improvement in the technology of gas recovery. Of course, the same could be applied to contamination of groundwater and soil.

### Estimating cancer incidence near incinerators

The basic formula to compute the number of cancer cases attributable to an incinerator is:

where *AC *= the attributable cancer incidence

*Rate_unex _*= background incidence rate in the general population

*ER *= excess risk in the exposed population (relative risk - 1)

*Pop_exp _*= number of exposed people

We assumed that the excess risk is not constant over time, but varies for a specific individual of the population at a given age and specific time as a function of various characteristics: level of cumulative exposure, latency since first exposure and latency since cessation of exposure (if any). We therefore assumed a theoretical model of cancer occurrence and imputed the varying excess risk around different incinerators, as a function of the different characteristics of the plant and of the nearby population. The methods are fully described in the additional file 1 (Appendix 1). Briefly, we modified the excess risk for overall cancer incidence estimated by Elliott et al. [14] (i.e. 3.5% for people exposed at incinerators operating before 1980, assuming 20 years of exposure) as a function of cumulative exposure (with exposure coefficients varying with time), latency since first exposure and latency since cessation of exposure. This algorithm was applied to the estimated 2001 population (by gender and age) living within 3 km of each specific incinerator to estimate the number of excess cancer cases in 2001-2050 attributable to exposures before 2001 and during 2001-2020. In Appendix 1, we illustrate the basic assumptions and we show how the excess risk during the 2001-2050 evaluation period varies in relation to time since the start of the operation of the plant and the time since cessation. The key assumption we made is that after 1980, due to technological improvements and as a result of national and European laws, the emissions from incinerators were reduced. For instance, measured particulate matter emissions from one incinerator in Italy (Modena) were 0.19 g/s in 1980-1989 (two lines), 0.0347 and 0.0376 g/s in 1995-1996 (two lines), 0.0196, 0.0273 and 0.104 g/s (three lines) in 1997-2002, and 0.0081, 0.0101, and 0.013 g/s (three lines) in 2003-2006. On the other hand, emission limits in the UK were reduced through legislation from 460 mg/m^3 ^(1968) to 200 mg/m3 (1983) to 30 mg/m^3 ^(1989/1990) and finally to 10 mg/m^3 ^in 2000. Based on these data, we assumed that if the exposure level was 1 before 1980, it was 0.8 in 1980-1989, 0.2 in 1990-2000, and 0.05 after 2000. In other words, we are assuming that the exposure levels during the eighties were somewhat lower (0.8) than during the seventies, during the nineties were fourfold lower, and in more recently they were twentyfold lower than in the seventies. We are highly confident about the scores we gave the exposure levels, as they are confirmed from measured data and are reflected in the legislation. Overall, we have a moderate level of confidence in the estimates of cancer cases, mainly due to the uncertainty characterizing the excess risk used.

### Estimating years of life lost (YoLL)

Assuming that chronic effects may continue to manifest themselves until 2050 in the entire population living close to incinerators in the three countries in 2001, and that their mortality rate was similar to that of the national population in 2001, we estimated Years of Life Lost attributable to PM_10 _and NO_2 _exposure as derived from the air dispersion model. In particular, we assumed that the impact of PM_10 _and NO_2 _was that which occurs during 2001-2020. As was done by Miller [26], we assumed constant future birth rates, constant hazard rates over time, immediate mortality effects after change in population-weighted exposure (no lag). The effects are calculated up to the year 2050 and all over a whole life span (105 years).

Overall, we have a high level of confidence in the estimates of YoLL, mainly due to the rather stable and well-established coefficients for NO_2 _and PM_10_.

### Estimating congenital anomalies and low birth weight near landfills

With a moderate level of confidence, we assumed that the only health impacts on populations living near landfills are congenital malformations and low birth weight. Other possible health effects were not considered, as our review of the literature did not reveal any other significant health effects. As already indicated, the time of the evaluation was 2001-2030 on the assumption that gas emissions (methane, carbon dioxide, non-methane volatile organic compounds, hydrogen sulfide and ammonia) from landfills last several years even after a landfill closure (we assumed 30 years). We cannot exclude that the effect on reproductive health occurs via groundwater or soil contamination. The formula to calculate the cases of malformation and babies of low birth weight attributable to residence near landfills is the same as for cancer incidence, where incidence should be changed with prevalence at birth and the number exposed are newborns.

Overall, we have a moderate level of confidence in the estimates of attributable cases of congenital anomalies and low birth weight, mainly due to the uncertainty in characterizing the excess risk ratios.

## Results

### Waste generation and management

Table 1 illustrates the basic statistics of waste management in Italy, Slovakia and England in 2001. The amount of MSW produced in Italy in 2001 was 31.94 million tonnes (Mtonnes), which corresponds to 560 kilograms per inhabitant. About 56% of Italian MSW was directed to landfills; recycling and composting accounted for 16% and 8% of MSW. For Slovakia, data on MSW production and management were available for 2002. The amount of MSW produced in that year was 1.52 million tonnes, which corresponds to 283 kilograms per inhabitant. From this total, 12% (0.18 Mtonnes) was recovered/treated (2.4% recycled, 2.6% composted, 6% recovered as energy and 1% treated by other methods) and 82% was disposed of (4.3% incinerated, 78.2% landfilled and 5.5 disposed by other methods). The amount of MSW produced in England was 28.8 million tonnes (Mtonnes), which corresponds to 587 kilograms per inhabitant. The majority of the MSW was landfilled (22 Mtonnes, or 77%), followed by recycling and composting (3.7 Mtonnes, 13%) and 9% of MSW was incinerated (2.6 Mtonnes).

**Table 1:** Waste Generation and Management in Italy, Slovakia and England in 2001.

Outcome	Italy		Slovakia		England	
	
	Thousands tons	%	Thousands tons	%	Thousands tons	%
Landfill	17910	56	1192	78	22180	77
Incineration	2590	8	65	4	2590	9
Recycled/composted	7650	24	76	5	3740	13
Other	3790	12	191	13	290	1
**Total**	**31940**	**100**	**1524**	**100**	**28800**	**100**
MSW generation per capita/kg	560		283		587	

### Population living close to incinerators and landfills

Table 2 shows the characteristics of the populations living close to the incinerators in the three countries. In Italy, more than a million people were included, with 9,010 newborns; less affluent social classes were over-represented compared to the national reference (25% in class V (deprived) and 12.6% in class I (less deprived), where classes are quintiles). A total of 64.4% of residents within 3 km were located in the 2-3 km band zone. Only 16,000 people lived close to the two incinerators in Slovakia. Also in this case, most of the residents lived in the farthest away circle. Contrary to Italy, the social class distribution around the two plants in Slovakia was skewed toward a higher social class. In England, approximately 1,200,000 people lived around the 11 incineration plants, mostly in the 2-3 km circular zone, and the social class distribution was strongly skewed towards deprivation (55% in class V (deprived) versus 3% in class I (less deprived).

**Table 2:** Characteristics of residents living within 3 km of an incinerator in Italy, Slovakia and England, 2001.

Variables	Italy		Slovakia		England	
	**N**	**%**	**N**	**%**	**N**	**%**

**Total**	1060569		16409		1203208	
**Sex**						
males	511831	48.3	8039	49.0	592817	49.3
females	548738	51.7	8370	51.0	610391	50.7
**Age (years)**						
0	9010	0.8	176	1.1	16425	1.4
1-14	123061	11.6	2914	17.8	233047	19.4
15-44	435825	41.1	7795	47.5	569850	47.4
45-64	289430	27.3	4337	26.4	229133	19.0
65+	203243	19.2	1187	7.2	154753	12.9
**Area-based socioeconomic status**						
I (high)	133211	12.6	9127	55.6	35498	3.0
II	159735	15.1	386	2.4	76359	6.3
III	223059	21.0	1600	9.8	150253	12.5
IV	257009	24.2	4856	29.6	274692	22.8
V (low)	264401	24.9	414	2.5	666406	55.4
missing information	23154	2.2	26	0.2		0.0
**Period of start of the plant**						
1960-1970	81586	7.7	0	0.0		0.0
1971-1980	127750	12.0	14240	86.8	533915	44.4
1981-1990	301950	28.5	2169	13.2		0.0
1991-2001	549283	51.8	0	0.0	669293	55.6
**Distance from the plant**						
0-1 Km	50990	4.8	221	1.3	95179	7.9
1-2 km	326798	30.8	3433	20.9	416987	34.7
2-3 km	682781	64.4	12755	77.7	691042	57.4

Table 3 shows the characteristics of the population living close to landfills in the three countries. The statistics for Italy were calculated for 118 sites in five regions and then extrapolated to the national level that included 619 sites. In Italy, more than 1,350,000 people were included (11,766 newborns); the social class distribution was skewed towards more deprivation (26% in class V (deprived) versus 13% in class I (less deprived)). The majority of residents within 2 km (85.7%) were located in the 1-2 km circular zone. A total of 328,869 people lived close to the 121 landfill plants in Slovakia. Also in this case, most of the residents lived in the longest circle farther away. In England, a total of 1,425,350 people lived close to the 232 geocoded landfills (including 16,242 newborns), especially in the 1-2 km circular area, and the social class distribution was skewed towards deprivation (20% in class V (deprived) versus 2.5% in class I (less deprived)), although the interpretation is difficult for the large proportion of the population with missing data on socioeconomic status.

**Table 3:** Characteristics of residents living within 2 km of landfills in Italy, Slovakia and England 2001.

	Italy observed data*		Italy estimated data**		Slovakia		England	
**Total**	257513		1350852		328869		1425350	
**Sex**								
Males	125750	48.8	659655	48.8	159822	48.6	694137	48.7
females	131763	51.2	691197	51.2	169047	51.4	731213	51.3
**Age (years)**								
0	2243	0.9	11766	0.9	3285	1.0	16242	1.1
1-14	32801	12.7	172066	12.7	59450	18.1	260043	18.2
15-44	107244	41.6	562577	41.6	156109	47.5	580430	40.7
45-64	67971	26.4	356560	26.4	76617	23.3	344290	24.2
65+	47254	18.4	247883	18.4	33408	10.2	224345	15.7
**Area-based socioeconomic status**								
I	34252	13.3	179678	13.3	79591	24.2	35277	2.5
II	38715	15.0	203090	15.0	81172	24.7	254972	17.9
III	57801	22.4	303210	22.4	74349	22.6	266629	18.7
IV	59320	23.0	311179	23.0	53893	16.4	271786	19.1
V	67339	26.1	353244	26.1	39855	12.1	286964	20.1
missing information	86	0.0	451	0.0	9	0.0	309722	21.7
**Distance from the plant**								
0-1 Km	36716	14.3	192603	14.3	59522	18.1	216938	15.2
1-2 km	220797	85.7	1158249	85.7	269347	81.9	1208412	84.8

*Only 118 out of 619 landfills were geocoded for Italy; ** estimation is based on data from the 118 landfills

Table 4 shows the results of the application of the local air dispersion model. Population-weighted additional exposure to PM_10 _and NO_2 _in 2001 is shown together with standard deviation and percentiles. The estimates for Italy and England derive from models with measured emissions (1st method) or national limits (2^nd ^method). The additional contribution to PM_10 _(using the national limit value) is 0.0114 μg/m^3 ^for Italy, 0.0078 μg/m^3 ^for Slovakia, and 0.0143 μg/m^3 ^for England. The additional contribution to NO_2 _(using the national limit value) is 0.2271 μg/m^3 ^for Italy, 0.1542 μg/m^3 ^for Slovakia, and 0.2855 μg/m^3 ^for England. The use of measured emission values had a strong impact on the estimate for PM_10 _(eg. 0.0030 μg/m^3 ^for Italy) but a lower impact for NO_2 _(e.g. 0.1944 μg/m^3 ^for Italy).

**Table 4:** Results of the application of the local air dispersion model for PM_10 _and NO_2 _around 40 incinerators in Italy, 2 incinerators in Slovakia and 11 incinerators in England.

	Italy	Slovakia	England
**PM_10 _(measured data)**			
Mean (SD)	0.0030 (0.0040)	na	0.0016 (0.0010)
25% percentile	0.0010	na	0.0009
50% percentile	0.0016	na	0.0014
75% percentile	0.0032	na	0.0020
			
**PM_10 _(national limits)**			
Mean (SD)	0.0114 (0.0151)	0.0078 (0.0037)	0.0143 (0.0083)
25% percentile	0.0038	0.0066	0.0084
50% percentile	0.0061	0.0075	0.0124
75% percentile	0.0120	0.0082	0.0181
**NO_2 _(measured data)**			
Mean (SD)	0.1944 (0.2583)	na	0.1346 (0.1056)
25% percentile	0.0658	na	0.0602
50% percentile	0.1050	na	0.1040
75% percentile	0.2060	na	0.1807
**NO_2 _(national limits)**			
Mean (SD)	0.2271 (0.3018)	0.1542(0.0747)	0.2855 (0.1666)
25% percentile	0.0769	0.131	0.1690
50% percentile	0.1220	0.149	0.2490
75% percentile	0.2400	0.163	0.3612

Population-weighted exposure to PM_10 _and NO_2 _in 2001 (data in μg/m^3^).

na: data not available

When PM_10 _and NO_2 _population-weighted exposure levels were examined by selected population characteristics, no differences were found for gender and age but higher exposure values were found among those of lower socioeconomic status in Italy and England (not in Slovakia) (data not shown).

### Health impacts due to incinerators

Table 5 shows the estimated number of additional cancer incident cases in the three countries for the period 2001-2050 as a result of exposure before 2001 (past exposure) and during 2001-2020 (current exposure). In Italy, an additional number of approximately 90 cases per year will be attributable to past exposure up to 2020 and then the number will decline to a minimum of 1.6 in 2050. On the other hand, the annual number of cases due to current exposure increases to 11 in 2020 and then will decline to 0 in 2050. In total, 2,729 additional cancer cases will be attributable to incinerators in Italy during 2001-2050 and the vast majority of them will be due to exposure before 2001. The total number of cancers attributable to exposure during 2001-2020 is 189.

**Table 5:** Estimated number of additional cancer cases in the three countries as result of exposure to incinerators before 2001 (past exposure) and during 2001-2020 (current exposure).

	Italy		Slovakia		England	
	
	Additional cases	95% CI	Additional cases	95% CI	Additional cases	95% CI
**Annual cases due to exposure before 2001 (Past exposure)**						
2001	88	76 - 101	0.82	0.71 - 0.94	33	28 - 38
2010	92	79 - 105	0.85	0.73 - 0.98	36	31 - 41
2020	89	76 - 101	0.84	0.72 - 0.96	36	31 - 41
2030	28	24 - 32	0.28	0.24 - 0.32	12	10 - 13
2040	2.0	1.4 - 2.6	0.002	0.001 - 0.002	0.068	0.058 - 0.077
2050	1.6	1.1 - 2.1	0	0 - 0	0	0 - 0
2001-2050*	**2540**	**2172-2896**	**23**	**20 - 27**	**1005**	**861-1149**
**Annual cases due to exposure during 2001-2020 (Current exposure)**						
2001	0	0 - 0	0	0 - 0	0	0 - 0
2010	2.7	2.3 - 3.1	0.017	0.015 - 0.020	1.7	1.5 - 2.0
2020	11	10 - 13	0.071	0.061 - 0.081	7.1	6.1 - 8.1
2030	4.6	4.0 - 5.3	0.029	0.025 - 0.033	2.9	2.5 - 3.3
2040	0.051	0.044 - 0.05858	0	0 - 0	0.032	0.028 - 0.037
2050	0	0 - 0	0	0 - 0	0.0	0 - 0
2001-2050*	**189**	**162-216**	**1.2**	**1.0 - 1.4**	**120**	**103 - 137**
**Total (Past + Current exposure)**						
2001	88	76 - 101	0.82	0.71 - 0.94	33	28 - 38
2010	95	81 - 108	0.87	0.75 - 1.0	38	33 - 43
2020	100	86 - 114	0.91	0.78 - 1.0	43	37 - 49
2030	33	28 - 37	0.31	0.026 - 0.035	15	13 - 16
2040	2.1	1.4 - 2.7	0.002	0.001 - 0.002	0.100	0.086 - 0.114
2050	1.6	1.1 - 2.1	0	0 - 0	0.0	0 - 0
2001-2050*	**2729**	**2334-3112**	**24**	**21 - 28**	**1125**	**964-1286**

* Cumulative number of cases during the period 2001-2050

In Slovakia, less than one additional case per year is estimated for past exposure during the whole period whereas the estimate for current exposure is very low. In total, 24 additional cancer cases will be attributable to incinerators in Slovakia during 2001-2050 and the majority of them will be due to exposure before 2001. The total number of cancers attributable to exposure during 2001-2020 is 1.2.

In England, an additional number of approximately 36 cases per year will be attributable to past exposure up to 2020 and then the number will decline to 0 in 2050. On the other hand, the annual number of cases due to current exposure increases to 7 in 2020 and then will decline to 0 in 2050. In total, 1,125 additional cancer cases will be attributable to incinerators in England during 2001-2050 and the vast majority of them will be due to exposure before 2001. The total number of cancers attributable to exposure during 2001-2020 is 120.

Table 6 shows the total number of Years of Life Lost, YoLL (also the YoLL per 100,000 inhabitants and the number of lost days per person) in the three countries attributable to exposure to PM_10 _or NO_2 _from incinerators. In Italy, the impact is higher for NO_2 _(total YoLL 3,621, 341.4 per 100,000 inhabitants) than for PM_10 _(total YoLL 181, 17.16 per 100,000 inhabitants). In Slovakia, the total number of YoLL is also higher for NO_2 _(37, 226 per 100,000) than for PM_10 _(2, 12.2 per 100,000). Comparable results were available for England with a total impact similar to Italy (for NO_2_: total YoLL 3,966, 330 per 100,000 inhabitants; for PM_10_: total YoLL 199, 16.5 per 100,000 inhabitants). Overall, the maximum impact of incinerators is 1.25 days per each person in Italy, 0.82 days per person in Slovakia, and 1.20 days per person in England.

**Table 6:** Estimated number of Years of Life Lost (YLL) (follow up to 2050) in the three countries as result of exposure to PM_10 _and NO_2 _from incinerators.

	Italy			Slovakia			England		
	
	Total YLL	YLL/100000	days/person	Total YLL	YLL/100000	days/person	Total YLL	YLL/100000	days/person
**PM_10 _(measured data)**	5	0.47	0.002	n.a	n.a	n.a	22	1.83	0.007
**PM_10 _(national limits)**	181	17.16	0.06	2	12.19	0.04	199	16.54	0.06
**NO_2 _(measured data)**	3099	292.2	1.07	n.a	n.a	n.a	1871	155.5	0.57
**NO_2 _(national limits)**	3621	341.4	1.25	37	225.5	0.82	3966	329.6	1.20

n.a.: not available

### Health impacts due to landfills

Table 7 shows the health effects of landfills in the three countries as annual cases of congenital malformations and newborns of low birth weight during the period 2001-2030. It is expected that an average of 1.96 (95%CI = 0.98-2.94) additional cases of birth defects per year occur in Italy, 1.54 (0.77-2.31) in Slovakia and 2.7 (1.35-4.0) in England. The estimated number of infants of low birth weight is 42 (95%CI = 35-42), 13 (11-13), and 58 (49-58) cases per year for 30 years, respectively for the three countries.

**Table 7:** Estimated health effects of exposures to landfills in the three countries as annual cases of congenital malformations and newborns of low birth weight.

	Italy			Slovakia			England		
	
	Expected cases	Additional cases	99% CI	Expected cases	Additional cases	99% CI	Expected cases	Additional cases	99% CI
All congenital anomalies	73	1.47	0.73 - 2.20	77	1.54	0.77 - 2.31	83	2.7	1.35 - 4.05
Neural tube defects	6	0.37	0.06 - 0.74	2	0.11	0.02 - 0.23	5	0.31	0.05 - 0.62
Hypospadias and epispadias	10	0.67	0.38 - 1.06	7	0.48	0.27 - 0.75	16	1.13	0.65 - 1.78
Abdominal wall defects	2	0.08	-0.09 - 0.24	12	0.60	-0.72 - 1.92	5	0.27	-0.32 - 0.86
Gastroschisis and exomphalos	2	0.27	0.05 - 0.51	12	2.16	0.36 - 4.09	5	0.85	0.14 - 1.61
Low birth weight	706	42.4	35.3-42.4	212	12.7	10.62 - 12.74	975	58.5	48.7 - 58.5

## Discussion

We found that the three countries differed with regard to recycling, landfilling and incineration policies: in Slovakia and England landfills were the most important method of management whereas Italy had a higher proportion of recycling and use of mechanical and biological treatment (MBT) technologies; incineration was used equally in Italy and England. There was a sizeable population living close to management plants in the three areas (e.g. approximately 2% of the entire population in Italy live close to an incinerator while an additional 2.5% live close to a landfill). In both Italy and England, populations of lower socio-economic status were more likely to live closer to waste disposal sites.

A systematic review of the scientific literature [4] revealed that cancer incidence and adverse reproductive outcomes (congenital malformations and low birth weight) are the main health effects possibly related to incinerators and landfills, respectively. On the basis of the relative risks derived from published data, we found that the largest health impact from incinerators during the period of evaluation (2001-2050) was cancer incidence accounting for a small percentage increase over the background in the exposed population. The majority of the cancer cases are due to exposures occurring before 2001 whereas the relative impact from the current exposure pattern is smaller. The health burden is thus not amenable to intervention from new policies since those cancer cases will occur in any case. On the other hand, policies for future developments should consider that most of the health effects will be seen over several decades.

Confirming preliminary research in the UK [27], the additional contribution to the PM_10 _and NO_2 _background in proximity of incinerators estimated with air dispersion models is relatively small and roughly equivalent in the three countries. The application of the air dispersion model data to a life table analysis indicates that the maximum impact of incinerators on the overall mortality of the resident cohort will be from exposure to NO_2_. A few hundred Years of Life Lost per 100,000 people over the period 2001-2020 are expected to occur and the results are consistent in the three countries. However, the burden estimated with a large scale model for the entire European population should be added to the overall impact of incineration as the impact is widespread.

Our evaluation of the impact of landfills is driven from the relative lack of scientific knowledge related to health effects since only adverse reproductive disorders were considered. The studies reviewed and used for impact assessment are based on distance from the sources and they do not provide indication of the relevant etiological exposures. For incinerators, there was a variety of emissions from the stacks of these plants, including particles and gases, Polycyclic Aromatic Hydrocarbons, heavy metals and dioxins for which a link with cancer can be easily justified. On the other hand, a biological explanation for the health impacts from landfills is more difficult since the causative agents (e.g. heavy metals, Polycyclic Aromatic Hydrocarbons, solvents) and the exposure routes (inhalation, ingestion of drinking water, contact with contaminated soil) for reproductive outcomes have not been indicated. Without identifying the causative agent/s and a plausible mechanism of effect and exposure, the level of confidence of the health impact assessment is moderate. Overall, the overall estimated burden in each country consists of few cases of congenital malformations and low birth weight newborns.

There are several examples in the literature of risk assessment of a single or a limited number of waste management plants [27]. Results of risk assessment performed at the country level are more limited, although the ExternE methodology [28,29] has been applied to estimate external costs of waste management. Rabl et al. [29] concluded that the only significant contributions come from direct emissions (of the landfill or incinerator) and from avoided emissions due to energy recovery (from an incinerator). Damage costs for incineration range from about 4 to 21 EUR tonne waste, and they are extremely dependent on the assumed scenario for energy recovery. For landfills the costs range from about 10 to 13 EUR tonne waste; it is dominated by greenhouse gas emissions because only a fraction of the CH4 can be captured. A complete assessment has been conducted in Singapore [30] but the main focus was on the environmental impact. Experiences of the health impact assessment in Europe are available from Ireland [31] and England [32]. The latest study provides a wide-ranging review focused on the environmental and health effects of MSW management.

Since lower socio-economic status is already associated with a higher risk of various negative health outcomes, an issue of environmental justice is present here because of the higher probability of exposure for less affluent people and their increased vulnerability. The situation is different for the two incinerators in Slovakia since they have an urban location and people living in urban areas in that country tend to have a higher socioeconomic profile. The issue has been extensively discussed in a recent paper [33] concluding that more effort should be made to investigate whether disadvantaged people are more vulnerable, i.e. risks differ in different social groups living in the same area. Notwithstanding this open question, decision makers should identify waste management policies that minimize their potential health impacts and unequal distribution.

### Limitations and uncertainties

In our study, we assessed the potential impact of incinerators and landfill sites on the health of the nearby population. However, there are some key choices and limitations that are important to consider. There are substantial environmental emissions associated with waste transport that we did not consider in the present assessment. In addition, it was outside the scope of the present study to consider the potential health effects of composting and of mechanical and biological plants, the two main alternatives to incineration and landfilling [3]. Finally, we did not take into account the potential occupational health risks to waste management workers, in particular occupational accidents.

Our health impact assessment is characterized by a number of uncertainties that are typical of these exercises focusing on the long term-effects of prolonged, low-level exposures. We have listed the sources of uncertainties for each step of our evaluation and briefly summarize our confidence in the methods and results. The direction of the bias generated by these uncertainties is unknown.

### Waste generation and management

As expected, there were inadequacies in data availability and reliability on MSW indicators as they are not uniform and not always available in the same format from published statistics. There were approximations in the available information on waste composition, classification of wastes is different in different countries, and we had high uncertainty concerning the amount and treatment of illegally disposed of waste. Overall, however, we have high confidence in the summary statistics that we have used and reported as they were available from reliable sources.

### Population characteristics and exposure to air pollutants

While we had relatively high quality data for incinerators in the three countries, exact coordinates of landfills were difficult to find in Italy. In addition, we did face difficulties in estimating the exposed population because the location of the plant was approximate, the size of some landfills is not known, and the unit of the available population data (census block) does not fit our needs. Population data by age and gender, available locally, are based on census and projections for years beyond the census. Overall, we have very high confidence in the population data close to incinerators but our confidence in population data close to landfills is only moderate.

The results of the air dispersion models depend on the quality of the input data. We had operational data measured during recent years for some of the incinerators but only estimated emissions for some others. In addition, some plant characteristics were missing and had to be imputed. On the other hand, we could rely on high quality meteorological data for most of the plants and topography was also considered. Overall, we have a moderate confidence in the estimated air pollution concentrations close to incineration plants.

### Relative risks and exposure-response functions

The application of excess-risk estimates based on distance from the plants has been problematic because of several difficulties in interpreting epidemiological studies. We have tried to address the issue in a transparent way by conducting a systematic evaluation, however, as underlined on several occasions above, we have moderate confidence in the excess risks used for the impact assessment of cancer cases and adverse reproductive outcomes. On the other hand, we have high confidence in the coefficients for long-term effects of PM_10 _and NO_2 _on mortality.

### Quantification of the health impact

The quantification has been straightforward in terms of calculating excess cases as there are no difficulties in finding the appropriate health statistics and in taking into account the particular population characteristics near the facilities. However, the most difficult part is attributing the effect studied from old plants using old technologies to new facilities. We tried to evaluate the consequence of changing some of the parameters. Overall, we have moderate confidence in our method to estimate excess cancer cases and reproductive outcomes. On the other hand, we think the life table approach is rather robust, despite the assumptions made (time of the effect, stability of the population, constant mortality). Finally, there may be more health effects of living near waste facilities that were not considered for lack of suitable evidence. For example, a general loss of quality of life, stress, odours, poor perceived health, besides being important health endpoints per se, may also contribute to increases in morbidity and mortality. For all of these reasons, we have a moderate level of confidence in our quantification of the health impacts.

Our approach to evaluate the level of confidence deserves discussion. Explicitly addressing uncertainty is an important contribution of research because it clarifies what is known and unknown and thus stimulates further investigation. We rated the confidence we had according to a scheme adapted from the one used by the IPCC. The approach has been recently used to address the health impact of ultrafine particles in an expert elicitation process [34]. In this case, the likelihood of an effect of ultrafine particles on natural mortality was considered as moderate whereas the likelihood of and effect on asthma aggravation was considered vey high.

## Conclusions

Past exposures from incinerators were likely to cause a sizeable health impact, especially for cancer, in Italy and England. However, the current impacts of landfilling and incineration can be characterized as moderate when compared to other sources of environmental pollution, e.g. traffic or industrial emissions, that have an importance on public health. The main results of the present study should be viewed in relation to the present debate within the EU and the Member States on the main policy issues related to waste management. Questions remain on the efforts that individual countries should make to reduce the overall amount of waste, and the appropriate targets to be met for recycling. Although waste to energy is gradually replacing old mass incineration, open issues remain also over the extent to which such policies should be introduced, given the possible alternatives [3]. There are several uncertainties and critical assumptions in our assessment model that are typical of a complex problem. However, we believe that it provides some insight into the relative health impact attributable to waste incineration and landfilling and that the model could potentially be useful as part of more articulated assessments for evaluating waste policy options, identifying knowledge gaps, and providing a framework for future comparative risk assessment.

## List of abbreviations

EU: European Union; INTARESE: Integrated Assessment of Health Risks of Environmental Stressors in Europe; IPCC: Intergovernmental Panel on Climate Change; UK: United Kingdom; PM10: Particulate Matter; NO2: Nitrogen Dioxide; GIS: Geographic Information System; CH4: Methane.

## Competing interests

The authors declare that they have no competing interests.

## Authors' contributions

FF conceived of and designed the study, participated in the statistical analysis and drafted the manuscript. CB dealt with the GIS system and with exposure assessment. KdH performed the dispersion models, participated in the design of the study and drafted the manuscript. MKvK participated in the study conception, design, and uncertainty assessment. MM participated in the study conception and design. FM participated in the study conception and design. LP participated in the design of the study and collected environmental data. DP participated in the design of the study, to statistical analysis and drafted the manuscript. PP participated in the study conception and design. AR participated in the design of the study and collected environmental data. CAP participated in the study conception and design. DB was involved in coordination and interpretation. All authors have read and approved the final manuscript.

## Supplementary Material

Additional file 1 **"Estimating attributable cancer incidence around incinerators"**. rationale, methods, calculation and sensitivity analysis for the estimates of the attributable cancer cases around incinerators. Click here for file
