# Supplementary material for: Health impact assessment of waste management facilities in three European countries
Source: Environ Health. 2011 Jun 2;10:53. doi: 10.1186/1476-069X-10-53 (PMC3129292; doi:10.1186/1476-069X-10-53)
Supplement: Additional file 1 — "Estimating attributable cancer incidence around incinerators". rationale, methods, calculation and sensitivity analysis for the estimates of the attributable cancer cases around incinerators. [file 1476-069X-10-53-S1.DOC]

## APPENDIX 1

## Estimating attributable cancer incidence around incinerators

#### **Rationale**

1. The basic formula to compute the number of cancer cases attributable to an incinerator is:

AC = *Rate_unex_ * ER * Pop_exp_*

where *AC* = the attributable cancer incidence

*Rate_unex_* = background incidence rate in the general population

*ER* = excess risk in the exposed population (relative risk – 1)

*Pop_exp_* = number of exposed people

1. We have relative risks calculated only for an arbitrarily defined exposed population (e.g. in terms of distance from an incinerator, Elliott et al.1996). Although the possibility to inference causality from these studies is limited (due to limitations of the studies discussed above), these estimates are the unique starting point for our assessment.
2. Once we have assumed that there is a relationship between living near incinerators and cancer incidence, we may suspect that the excess risk is not constant over time, but varies for a specific individual of the population at a give age and specific time as a function of various characteristics: level of attained cumulative exposure, latency since first exposure and latency since cessation of exposure (if any).
3. We therefore need to assume a theoretical model of cancer occurrence and to impute the varying excess risk around different incinerators, as a function of the different characteristic of the plant and of the nearby population.

#### **Assumptions**

1. Model of carcinogenesis. We do not have clear scientific data about the carcinogenic model underlying the association between living close to the plants and occurrence of cancer. We may assume here that the model that better fit our purpose is the most studies one that relates cigarette smoking with lung cancer. Under the multistage theory of cancer proposed by Armitage and Doll (Armitage and Doll, 1958), Doll and Peto (1978) indicated that the excess relative risk of lung cancer is a function of attained age together with a complex dependency related to age at starting, duration and intensity of smoking and time since quitting. Various attempts have been made to validate the model using data from real long term cohorts (Hazelton et al. 2005; Schollnberger et al. 2006). Although the results of these studies do not provide a uniform response regarding the role of each factor (Hornsby et al. 2007), and the approach may be seen as a simplification, it has the advantage to provide a template for addressing other exposure-response relationships (Siemiatycki, 2005). It is clear that this model that is mostly applicable to solid cancers of epithelial origin. The approach could be different for hematological or soft tissues cancers or for childhood cancer. Finally, the model that we assume is multiplicative in nature, namely that the excess risk is a multiplicative function of the baseline risk.
2. Uniform excess risk in the area within 3 Km. We may assume that on a given year the excess risk cross all exposed areas around a given incinerator in the study (3 km) is equal to that derived from the scientific literature with corrections depending on several factors referenced above.
3. Reference Excess Risk (RER). We may assume as reference that the value of 3.5% (95%CI: 3-4%) excess risk reported in the paper by Elliott et al (1996) reflects the additional risk of total cancer incidence for a population living within 3 km from an incinerator exposed for a duration of 20 years at the levels of contamination that were present in the period 1960-1980. We can call this value Reference Excess Risk (RER). In fact, all the 72 incinerators studied in the Elliott’s paper did start operation before 1976, the follow-up was conducted during 1974-1986 (1974-1987 for Wales and 1975-1987 for Scotland), and the effect estimate was given considering 10 years of latency for solid cancers.
4. Exposure levels vary with time. We may assume that in subsequent years after 1980, due to technological improvements and as results of national and European laws, the emissions from incinerators have been reduced. For instance, measured particulate matter emissions from one incinerator in Italy (Modena) where 0.19 g/s in 1980-1989 (two lines), 0.0347 and 0.376 g/s in 1995-1996 (two lines), 0.0196, 0.0273 and 0.104 g/s (three lines) in 1997-2002, and 0.0081, 0.0101, and 0.013 g/s (three lines) in 2003-2006. On the other hand, emission limits in the UK were reduced through legislation from 460 mg/m3 (1968) to 200 mg/m3 (1983) to 30 mg/m3 (1989/1990) and finally to 10 mg/m3 in 2000. On the basis of these data, we can assume that if the exposure level was 1 before 1980, it was 0.8 in 1980-1989, 0.2 in 1990-2000, and 0.05 after 2000. In other words, we are assuming that the exposure levels during the eighties were lower (0.8) that during the seventies, during the nineties were fourfold lower, and in more recent times they were twentyfold lower that the seventies. Of course, these assumptions may be varied in sensitivity analysis.
5. Calculation of cumulative exposure. We need to recognise that at a given age of a person, the best way to summarize the exposure experience is to calculate cumulative exposure (CE) as the sum of the exposure contribution during the different periods. The analogues for cigarette smoking are pack-years. For example, a person aged 60 in 2001, living nearby an incinerator opened in 1980 and still running in 2001, will have over the period 1980-2001 a CE of 10.25 (8+2.2+0.05=10.25, i.e. 10 years at exposure 0.8 in 1980-1989, 11 years at exposure 0.2 in 1990-2000, and one year in 2001 at exposure 0.05).
6. Latency since first exposure and latency since exposure cessation. Finally, latency since first exposure is a relevant issue, especially if a long time for the evaluation is to be considered. For most solid cancers, there is some cancer expression only several years after first exposure to carcinogens and the full effect is appreciable only after 20 years (as indicated above, latency may be shorter for non solid cancers). In our case, we assume that the effect of the exposure to a given incinerator will be appreciable only after some years from first exposure, the peak will be reached after 20 years and it will be constant up to 40 years, then it will start to smoothly decline approaching 0 after 70-80 years. On the other hand, if the exposure is removed, as in the case of smoking cessation, the risk declines as a function of the time since cessation. We may assume that the excess risk will smoothly decline soon after cessation of exposure.
7. For practical reasons, we need to assume that the population selected on a given year has been always living close to the plant and it size and age composition will be constant during the period of the evaluation.

#### **Calculations**

1. Time and age. For a specific age class ( *a_i*) of the population we wish to consider, we define the time elapsed (*t_exp_)* from the start of exposure to the incinerator (ys) and the reference year (or year of calculation).

$t_{\exp_{a\_i}}=\min\left\{\frac{a(M)_{i}-a(m)_{i}}{2};y-ys\right\}$ (1),

where:

*a_i=i-th age class*

*a(M)i = max. age in i-th class*

*a(M)i = min. age in i-th class*

*y=reference year (or year of calculation)*

*ys= year of start*

Example: incinerator Modena (start in 1980), reference year : 2001, age class: 30-34 years

Then $t_{\exp_{a\_30-34}}=\min\left\{\frac{34+30}{2};\text{2001-1980}\right\}=\min\left\{32;20\right\}=20$

2. Cumulative exposure. For a given age class, cumulative exposure is given by the following formula:

$CE_{t_{\exp_{a\_i}}}=\sum_{t=1}^{t_{\exp_{a\_i}}} Ey(t)$ (2),

where *Ey* is the exposure factor for a given year according to the rule:

:

$Ey(t)=\left\{\begin{matrix} 1\text{ , t}<1980 \\ 0.8\text{ ,}1980\leq t<1990\text{ } \\ 0.2\text{ , 1990 }\leq t<2001 \\ 0.05\text{ , }t\geq\text{2001 } \end{matrix}\right.$ (3)

And shown in the graph below.


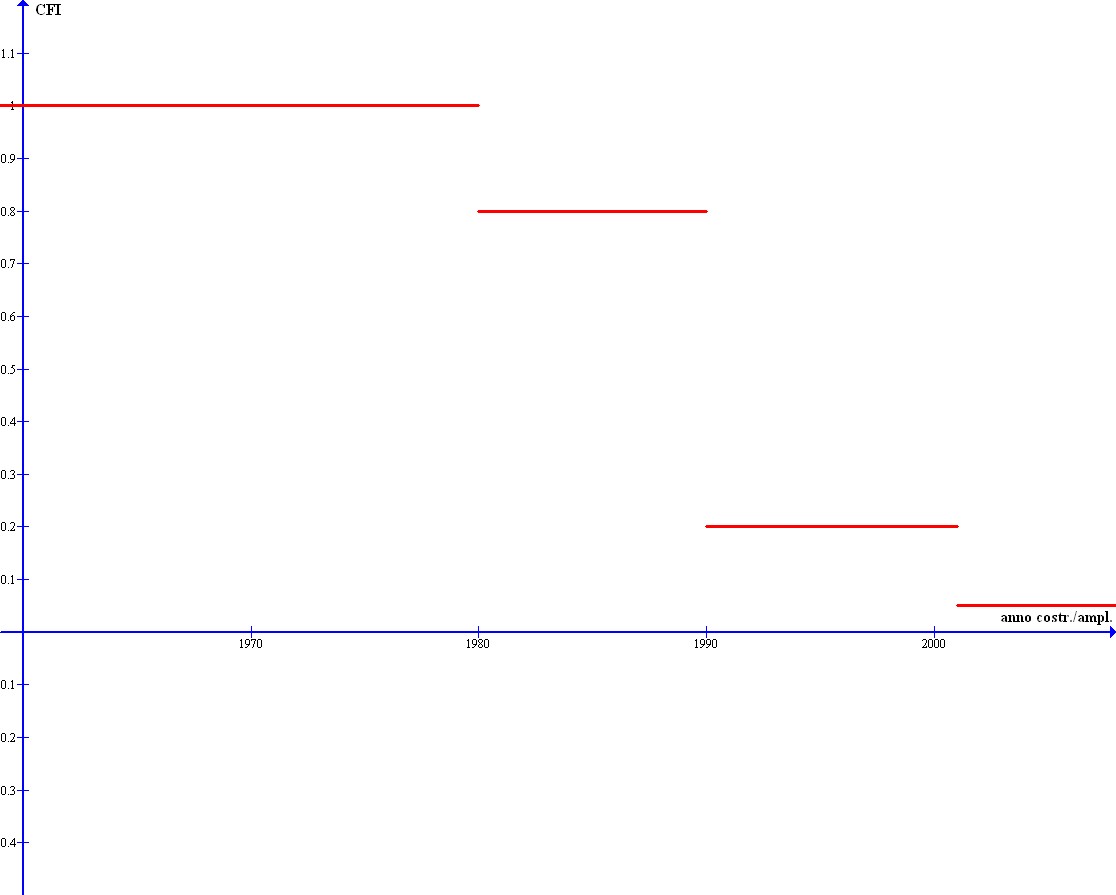


3. Latency since first exposure. We define then latency since start of exposure (Ls) for a given age class (a_*i* ) as a function of the time variable indicated above:

$Ls=f(t_{\exp_{a\_i}})=\left\{\begin{matrix} \frac{1}{1+b*e^{-ct_{\exp_{a\_i}}}}\text{ , }t_{\exp_{a\_i}}\leq40 \\ 1-\frac{1}{1+b*e^{-c(t_{\exp_{a\_i}}-40)}}\text{ , }t_{\exp_{a\_i}}>40 \end{matrix}\right.$ (4),

where:

*Ls= latency since first exposure*

*b and c= coefficients for a sigmoid curve that reaches the plateau (one) 20 years since first exposure, remains stable until 40 years, and then starts to decline reaching 0 after 80 years as indicated in the graph below.*


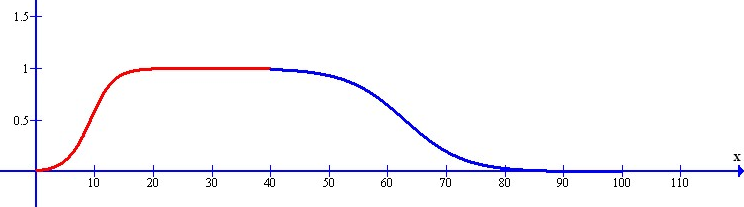


4. Latency since cessation of exposure. To allow for the possible effect of cessation of exposure, we assume a factor for latency since cessation of exposure (Lc) that follows a sigmoid with a decrease of the risk starting after the closure and reaching a plateau after 20 years as indicated in the graph below.


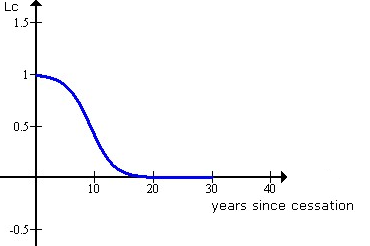


This factor follows the function below:

$Lc=f(t_{c})=1-\frac{1}{1+b*e^{-c(t_{c})}}\text{ }$ (5),

Where *tc is time since cessation of exposure*

For each age class and at a given time (year), the three factors indicated above (CE, Ls and Lc) act in a multiplicative way to modify the Reference Excess Risk (from Elliott et al. 1996).

Thus, for a given age class ( *a_i*):

$ER_{a\_i}=RER*(CE_{a\_i}/20)*Ls*Lc$ (6),

Where:

*ERa_i = the estimated excess risk of cancer incidence*

*RER =the reference excess risk as estimate from Elliott et al (1996) (3.5% increase for exposure of 20 years to incinerators operating before 1980).*

*CEa_i = cumulative exposure*

*Ls =latency since start of exposure*

*Lc =latency since cessation of exposure r*

Finally, for a given age class ( *a_i):*

$AC_{a\_i}=ER_{a\_i}*Rate_{un\exp}*Pop_{\exp}$ (7),

Where:

*ACa_i =attributable cancer incidence*

*ER a_i =excess risk of cancer incidence*

*Rate_unex_ =background incidence rate in the general population*

*Pop_exp_ =number of exposed people*

**Application of the model**

The figure below shows the results of the application of the model from 1960 to 2050. For each year, the excess risk (ER) (age weighted) of cancer is calculated with reference to a theoretical Italian population (age distribution) living close to an incinerator as function of year of starting operation.

The next figure illustrate the estimated excess risk for a population living close to a plant operating since 1980 as function of the year of closing. The excess risks are reported up to 2050.

**Sensitivity analysis**

In our model to estimate cancer incidence attributable to incinerators operating in 2001-2020, three assumptions are critical: the coefficient we assigned to the exposure level in 2001-2020, i.e. 0.05; the form of the relationship with latency since exposure began and the form of the relationship with time since exposure ceased. While the first assumption is related to emission control measures, the other two are connected with the biological relationship between exposure to carcinogens and occurrence of cancer. We modified these assumptions in order to perform a sensitivity analysis as follows.

1. We modified our original assumption that incinerators after 2001 emit twentyfold less air pollutants than old incinerators operating before 1980 with a more radical coefficient of 0.02 (fifty-fold reduction). The first graph below shows, for an incinerator operating since 1980, a slight change in the Excess Risk (ER, Y axis) after 2001.
2. We modified our original assumption that the full effect on cancer expression is reached 20 years since first exposure and has a plateau lasting up to 40 years. We assumed a faster slope of the latency function and a shorter duration of the plateau: the peak of the effect is already reached at 10 years and declines after 20 years. The second graph below shows, for an incinerator operating since 1980, a shift to the left in the Excess Risk (ER, Y axis) before 2001 and a rapid decline since that date.
3. We modified our original assumption that the effect declines smoothly after cessation of exposure, reaching a minimum after 20 years applying a stronger effect of time since cessation reaches a null effect 10 years after closure. The third graph below shows, for an incinerator operating since 1980, a shift to the left in the Excess Risk (ER, Y axis) after cessation of exposure.

**References**

Armitage P, Doll R: **The age distribution of cancer and a multi-stage theory of carcinogenesis**. *British Journal of Cancer* 1954, **8**:1-12.

Doll R, Peto R: **Cigarette smoking and bronchial carcinoma: dose and time relationships among regular smokers and lifelong non-smokers**. *Journal of Epidemiology and Community Health* 1978, **32**:303-313.

Elliott P, Shaddick G, Kleinschmidt I, Jolley D, Walls P, Beresford J, Grundy: **Cancer incidence near municipal solid waste incinerators in Great Britain**. *British Journal of Cancer.* 1996, **73**:702-710.

Hazelton WD, Clements MS, Moolgavkar SH. **Multistage carcinogenesis and lung cancer mortality in three cohorts**. *Cancer Epidemiology Biomarkers & Prevention* 2005, **14**:1171-81.

Hornsby C, Page KM, Tomlinson IP: **What can we learn from the population incidence of cancer? Armitage and Doll revisited**. *Lancet Oncolology* 2007, **8**:1030-1038.

Schöllnberger H, Manuguerra M, Bijwaard H, Boshuizen H, Altenburg HP, Rispens SM, Brugmans MJ, Vineis P: **Analysis of epidemiological cohort data on smoking effects and lung cancer with a multi-stage cancer model**. *Carcinogenesis* 2006, **27**:1432-1444.

Siemiatycki J: **Synthesizing the lifetime history of smoking**. *Cancer Epidemiology Biomarkers & Prevention*. 2005, **14**:2294-2295.
